# A review of clinical characteristics and genetic backgrounds in Alport syndrome

**DOI:** 10.1007/s10157-018-1629-4

**Published:** 2018-08-20

**Authors:** Kandai Nozu, Koichi Nakanishi, Yoshifusa Abe, Tomohiro Udagawa, Shinichi Okada, Takayuki Okamoto, Hiroshi Kaito, Katsuyoshi Kanemoto, Anna Kobayashi, Eriko Tanaka, Kazuki Tanaka, Taketsugu Hama, Rika Fujimaru, Saori Miwa, Tomohiko Yamamura, Natsusmi Yamamura, Tomoko Horinouchi, Shogo Minamikawa, Michio Nagata, Kazumoto Iijima

**Affiliations:** 1 Department of Pediatrics, Kobe University Graduate School of Medicine, 7-5-1 Kusunoki-cho, Chuo-ku, Kobe, 650-0017 Japan; 2 Department of Child Health and Welfare (Pediatrics), Graduate School of Medicine, University of the Ryukyus, Nishihara, Japan; 3 Children Medical Center, Showa University Northern Yokohama Hospital, Yokohama, Kanagawa Japan; 4 Department of Pediatrics and Developmental Biology, Tokyo Medical and Dental University, Tokyo, Japan; 5 Division of Pediatrics and Perinatology, Faculty of Medicine, Tottori University, Tottori, Japan; 6 Department of Pediatrics, Hokkaido University Graduate School of Medicine, Sapporo, Japan; 7 Department of Pediatrics, National Hospital Organization Chiba-East Hospital, Chiba, Japan; 8 Department of Pediatrics, Faculty of Medicine, University of Yamanashi, Kofu, Japan; 9 Department of Nephrology, Aichi Children’s Health and Medical Center, Obu, Japan; 10 Department of Pediatrics, Wakayama Medical University, Wakayama, Japan; 11 Department of Pediatrics, Osaka City General Hospital, Izumi, Japan; 12 Department of Pediatrics, The Jikei University School of Medicine, Tokyo, Japan; 13 Department of Pediatric Nephrology and Metabolism, Osaka Women’s and Children’s Hospital, Izumi, Japan; 14 Kidney and Vascular Pathology, Faculty of Medicine, University of Tsukuba, Tsukuba, Japan

**Keywords:** Genotype–phenotype correlation, Thin basement membrane, ACE inhibitor, Bardoxolone

## Abstract

Alport syndrome (AS) is a progressive hereditary renal disease that is characterized by sensorineural hearing loss and ocular abnormalities. It is divided into three modes of inheritance, namely, X-linked Alport syndrome (XLAS), autosomal recessive AS (ARAS), and autosomal dominant AS (ADAS). XLAS is caused by pathogenic variants in *COL4A5*, while ADAS and ARAS are caused by those in *COL4A3*/*COL4A4*. Diagnosis is conventionally made pathologically, but recent advances in comprehensive genetic analysis have enabled genetic testing to be performed for the diagnosis of AS as first-line diagnosis. Because of these advances, substantial information about the genetics of AS has been obtained and the genetic background of this disease has been revealed, including genotype–phenotype correlations and mechanisms of onset in some male XLAS cases that lead to milder phenotypes of late-onset end-stage renal disease (ESRD). There is currently no radical therapy for AS and treatment is only performed to delay progression to ESRD using nephron-protective drugs. Angiotensin-converting enzyme inhibitors can remarkably delay the development of ESRD. Recently, some new drugs for this disease have entered clinical trials or been developed in laboratories. In this article, we review the diagnostic strategy, genotype–phenotype correlation, mechanisms of onset of milder phenotypes, and treatment of AS, among others.

## Introduction

Alport syndrome (AS) is a progressive hereditary renal disease accompanied by sensorineural hearing loss and ocular abnormalities. AS develops because of pathogenic variants in the *COL4A3, COL4A4*, and *COL4A5* genes encoding type IV collagen α3, α4, and α5 chains, respectively [1–3]. These type IV collagens constitute the glomerular basement membrane (GBM). AS has been reported to develop in approximately one in 5000 people; it comprises 0.5% of newly developed end-stage renal disease (ESRD) cases in adults [4] and 12.9% in children [5]. Type IV collagen has six different α chains, α1 to α6, which construct triple helix structures in which the three chains are combined. The combination of three α-chains is organ-specific: in the GBM, cochlea basement membrane, and base of the ocular lens, the triplet α3–α4–α5 is present, while in Bowman’s capsule and skin basement membrane, it is α5–α5–α6. When an abnormality occurs in the α-chain, these triple helix structures are broken, causing nephropathy, sensorineural hearing loss, and eye lesions. AS is divided into X-linked Alport syndrome (XLAS), autosomal recessive AS (ARAS), and autosomal dominant AS (ADAS), according to its mode of inheritance. The distribution of these cases is reported to be as follows: 80% XLAS, 15% ARAS, and 5% ADAS. XLAS is caused by abnormality of *COL4A5*, while ADAS and ARAS are caused by abnormality in *COL4A3* or *COL4A4* [3].

## Clinical characteristics and diagnostic features

Table 1 shows the diagnostic features established by the members of the Working Group for Alport Syndrome in the Japanese Society of Pediatric Nephrology (JSPN) in 2015 [6]. The main criterion is persistent hematuria. When patients fulfill one or more secondary features, or two or more accessory features, in addition to the primary feature, they can be diagnosed with AS. Symptoms differ depending on the mode of inheritance, with their features described below.

**Table 1:** Diagnostic features of Alport syndrome (revised in February 2015) prepared by the Working Group on Alport Syndrome of the Japanese Society of Pediatric Nephrology

I. Primary feature:	
I-1. Persistent hematuria *1	

II. Secondary features:
II-1. Mutations in type IV collagen genes *2
II-2. Type IV collagen abnormal expression *3
II-3. Glomerular basement membrane (GBM) -specific abnormalities *4

III. Accessory features
III-1. Family history of kidney diseases
III-2. Bilateral sensorineural deafness
III-3. Ocular abnormalities *5
III-4. Diffuse leiomyomatosis

In addition to the primary feature, patients satisfy one or more secondary features

If there is no corresponding item in secondary features, patients should satisfy two or more of the accessory features

If patients have only the primary feature and have a family member diagnosed with Alport syndrome, the case is set as a “suspected case”

Asymptomatic carriers can be diagnosed with any one feature of type IV collagen (II-1 or II-2) among the secondary features

For all features, those caused by other diseases should be excluded, for example, a family history of kidney failure due to diabetes or senile deafness

*1 Persisted for more than 3 months, which was confirmed by urinalysis on at least two occasions. As a rare situation, there is a possibility that hematuria may disappear at the time when renal failure progresses to the end stage, in which case appropriate examination such as of kidney dysfunction should be performed

*2 This refers to a homozygous or heterozygous mutation of *COL4A3* or *COL4A4*, or a hemizygous (male) or heterozygous (female) mutation of the *COL4A5* gene

*3 Type IV collagen α5 chain exists not only in the glomerular basement membrane and Bowman’s capsule, but also in the skin basement membrane. Upon immunostaining using anti-α5-chain antibody, normal glomeruli and skin basement membrane are stained linearly and continuously. However, glomeruli, Bowman’s capsule, and the skin basement membrane of male patients with X-linked Alport syndrome are completely negative. In glomeruli, Bowman’s capsule and skin basement membrane of female patients are partially stained. In autosomal recessive Alport syndrome, the α3-, α4-, and α5 chains are not stained in glomerular basement membranes, whereas in Bowman’s capsule and skin, normal α5-chain staining is shown. Note that the above is a typical pattern, but an atypical pattern also exists. Moreover, Alport syndrome cannot be ruled out even if α5 chain expression shows completely normal pattern

*4 Glomerular basement membrane-specific abnormalities include broad irregular thickening of the glomerular basement membrane and reticulation of the lamina densa. Extensive thinning of the glomerular basement membrane frequently seen in benign familial hematuria is also seen in Alport syndrome, which can be the only finding of the glomerular basement membrane. In these cases, there is a high possibility of Alport syndrome if the cases show hearing loss, ocular abnormalities, or a family history of renal failure

*5 Specific ocular abnormalities include anterior lenticonus, posterior subcapsular cataract, posterior polymorphous dystrophy, and retinal flecks

1. XLAS

In XLAS, there is often a family history of hematuria (with or without proteinuria) or renal failure. However, in approximately 15% of cases, there are de novo variants without a family history [3]. Microscopic hematuria is observed in all male cases. In female patients, it is observed in approximately 98% with hematuria and 73% with both hematuria and proteinuria [7]. In male patients, proteinuria is recognized at an early stage of childhood, sometimes exhibiting a nephrotic status; it is also reported that 90% of patients develop ESRD by the age of 40 years, with the median age of development of ESRD being 25 years [8]. In females, 12% of cases have developed ESRD by the age of 40 [9]. We recently reported that patients exhibited proteinuria at a median age of 7 years, and that ESRD development occurred at a median of 65 years in female XLAS [7].

Sensorineural hearing loss often occurs from the latter stage of childhood; 90% of male patients and approximately 12% of female patients present with hearing loss by the age of 40 years [8, 9]. Jais et al. reported that, in females, the presence of hearing loss predicts the development of ESRD; however, our recent study showed no significant difference in kidney severity between patients with and without hearing loss [7, 9]. Specific ocular abnormalities, including anterior lenticonus, posterior subcapsular cataract, posterior polymorphous dystrophy, and retinal flecks, are known as ophthalmologic complications; clinical symptoms, such as obvious visual impairment, rarely appear [10]. Other complications include leiomyoma due to contiguous gene deletion encompassing the 5′ end of *COL4A5* and *COL4A6* intron 2. In this phenotype, leiomyoma is observed with full penetration, for which no gender difference is observed [11].

2) ARAS

Clinically, ARAS shows symptoms similar to those in male XLAS patients. There are no gender differences in clinical symptoms and incidence, and this condition sporadically occurs in one generation. Families with monoallelic variant carriers are often asymptomatic or show only microscopic hematuria (and mild proteinuria) [12, 13]. For the genetic diagnosis of ARAS, analysis of at least one familial member (ideally, both parents) is necessary to prove that two heterozygous variants are located in trans positions on two different alleles (either *COL4A3* or *COL4A4*). In terms of the clinical findings that we previously reported, the median age of ESRD development is 21 years. Sensorineural deafness was observed at a median age of onset of 20 years [12].

3) ADAS

Recently, we published an article regarding the clinical picture, pathology, and genetic background of ADAS [14]. The median age for detecting proteinuria was 17.0 years, and that for developing renal insufficiency was 70 years. In addition, both hearing loss and eye lesion were reported to occur quite rarely. Moreover, three of 16 patients with pathological findings showed focal segmental glomerular sclerosis (FSGS), as revealed by light microscopy. It has also been reported that, in approximately 10% of patients with familial focal segmental glomerulosclerosis, *COL4A3* or *COL4A4* mutations are identified, suggesting that there are many undiagnosed ADAS patients [15].

## Pathological findings

**Fig. 1:**
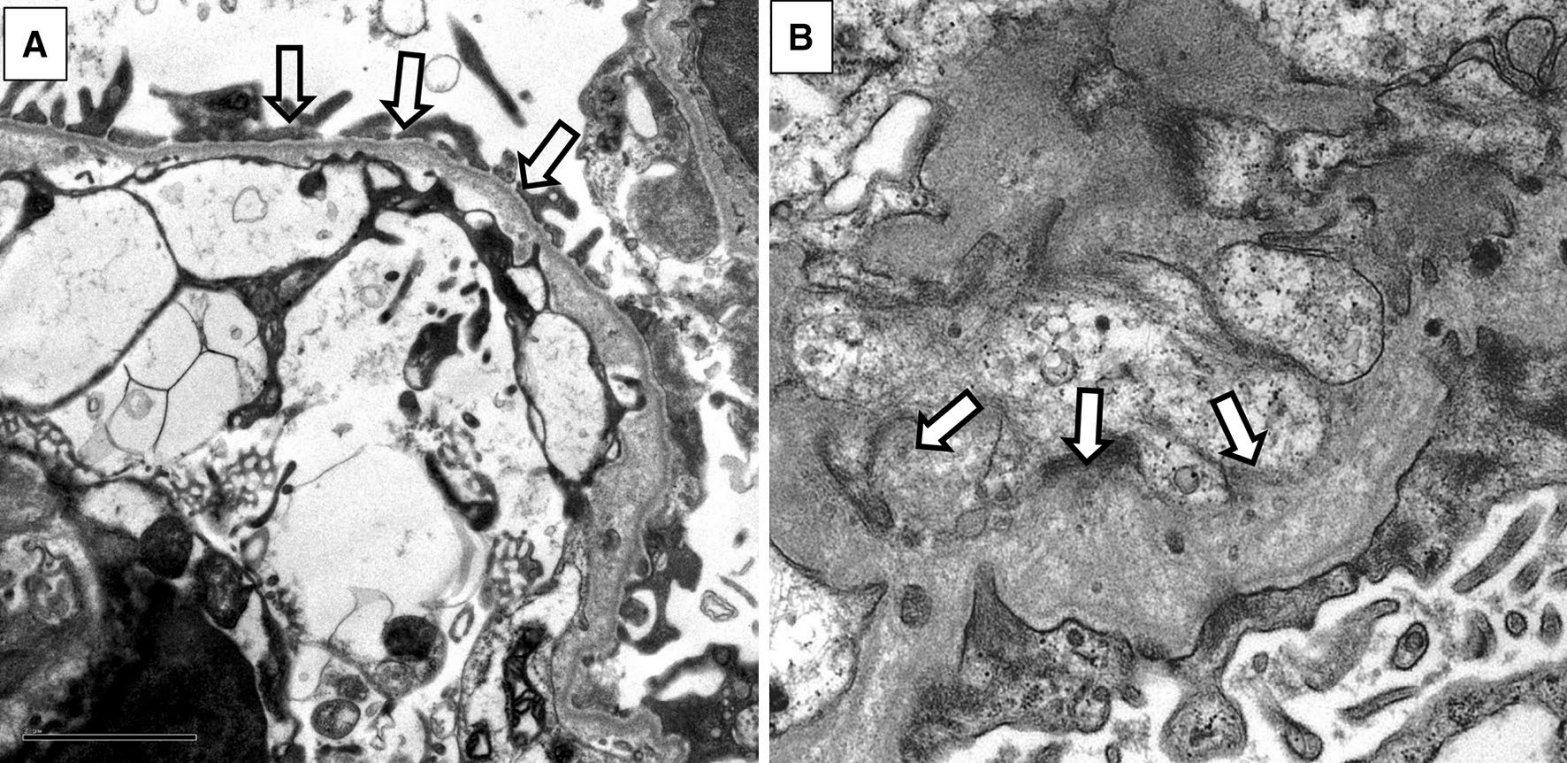
Glomerular basement membrane (GBM) change in Alport syndrome (AS) observed by electron microscopy. **A** Thin basement membrane, which is typically observed in milder cases, including female X-linked AS and autosomal dominant AS. **B** Diffuse thickening and lamellation, which are specific findings of AS

**Fig. 2:**
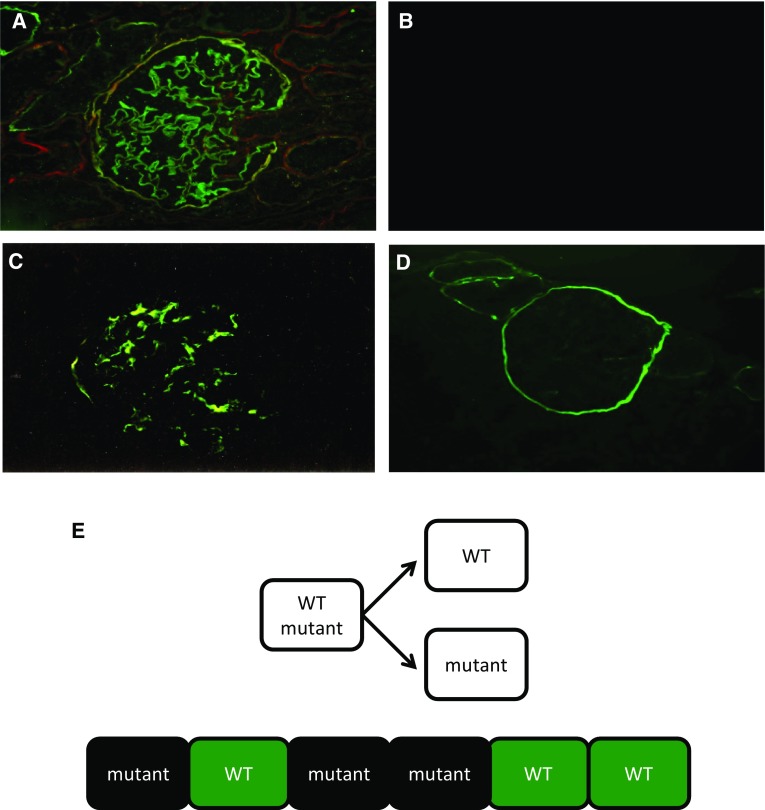
Immunohistochemical analysis of type IV collagen α5 chain in glomerulus. **A** Normal control shows full expression in both glomerular basement membrane (GBM) and Bowman’s capsule (BC). **B** Male X-linked Alport syndrome (XLAS) case shows completely negative expression in both GBM and BC. **C** Female XLAS case shows a mosaic pattern of expression in both GBM and BC due to the mechanisms of X-chromosome inactivation that occur in female cells. **D** Autosomal recessive Alport syndrome case shows negative expression only on GBM and positivity on BC, because BC consists of the α5–α5–α6 triple helix. **E** Schema for X-chromosome inactivation (XCI). In all female cells, either of the two X chromosomes is randomly inactivated. When the wild-type chromosome has been inactivated, the cell will not produce α5. Then, GBM and BC will be stained with a mosaic pattern

There are no specific light microscopic findings in AS; nonspecific findings are observed, such as mesangial proliferation, FSGS, renal tubular atrophy, foam cell formation, or interstitial fibrosis. Electron microscopic (EM) findings show irregular thickening and thinning of the GBM, and lamellation and splitting in lamina densa can be observed (Fig. 1). These findings are specific to AS and electron microscopic findings are indispensable for the pathological diagnosis of this condition. However, pathological findings become apparent in the form of nephritis progression, even in male XLAS cases or ARAS cases; for female XLAS and ADAS cases, typical findings of EM can be observed generally at later stages and often show only thinning of GBM. Therefore, careful observation is necessary, because there are cases with no obvious changes except for thin basement membrane (TBM) at the early stages of AS. Immunohistologically, specific findings can be identified with α5 staining. Approximately 80% of XLAS male patients are completely negative for α5 staining, while XLAS female patients exhibit a mosaic pattern due to the mechanisms of X-chromosome inactivation that occur in female cells (Fig. 2) [2, 16]. In ARAS patients, the GBM is negative for α3, α4, and α5 staining, while Bowman’s capsule is α5 positive; this is because the GBM consists of α3–α4–α5, but Bowman’s capsule consists of α5–α5–α6 and *COL4A3*/*COL4A4* mutations do not influence α5 expression in Bowman’s capsule. In ADAS patients, both the GBM and Bowman’s capsule exhibit a normal pattern of α5 expression. α5 staining is a useful diagnostic method, because it remains unchanged regardless of age; however, it should solely be used as a method of auxiliary diagnosis, because it can show nonspecific findings, such as a normal expression pattern in more than 20% of XLAS males [1]. We have reported that 29% of male XLAS and 20% of ARAS cases showed atypical positive expression of α5 in GBM [12, 17]. All male XLAS cases with α5 positivity possessed non-truncating variants or somatic mosaic variants in *COL4A5* and showed significantly milder phenotypes of later-onset ESRD [17]. ARAS cases with α5 positivity possessed a missense variant in at least one allele in the *COL4A3* or *COL4A4* gene and again showed a milder phenotype that involved later development of ESRD [12].

**Fig. 3:**
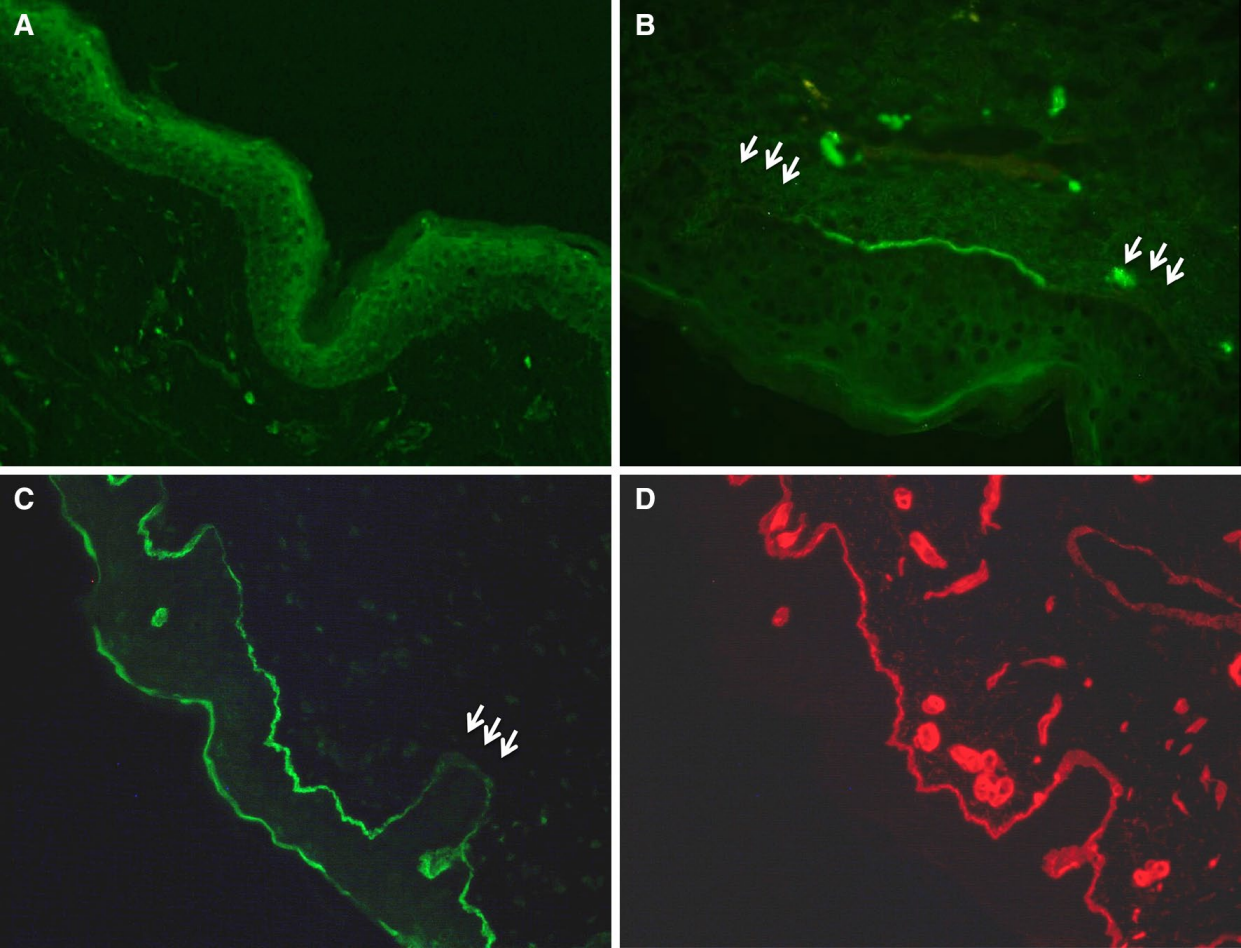
Immunohistochemical analysis of type IV collagen α5 chain on epidermal basement membrane (EBM). **A** Male X-linked Alport syndrome (XLAS) case shows completely negative expression on EBM. **B** Female XLAS case shows a mosaic pattern of expression on EBM due to the mechanisms of X-chromosome inactivation that occur in female cells. **C** Normal control shows full α5 expression on EBM; however, decreased α5 expression can be observed in the bottom of papillary EBM in the normal skin. **D** Normal control shows full α2 expression on EBM, even at the bottom of papillary EBM

Skin basement membrane consists of type IV collagen α5–α5–α6 triple helix. Therefore, α5 staining is a useful auxiliary diagnostic method only for XLAS cases: male XLAS cases show negativity for α5 expression, while female XLAS cases show a mosaic expression pattern, as observed in GBM (Fig. 3A, B) [2]. It should be noted that decreased α5 expression can be observed at the bottom of the papillary epidermal basement membrane in normal skin (Fig. 3C, D) [18].

## Gene tests

Along with the progression of gene analysis technology in recent years, there has been remarkable development of the genetic diagnosis system for AS. Because all three of the *COL4A3, COL4A4*, and *COL4A5* genes consist of approximately 50 exons, conventional Sanger sequencing is too laborious and time-consuming for genetic screening. Therefore, the main strategy for gene screening in AS has become targeted sequencing, which captures all exons and exon–intron boundaries of all three genes, along with comprehensive sequencing analysis by NGS. Genes responsible for kidney diseases that clinically or pathologically resemble AS should be included in the targeted screening panel. For example, BOR syndrome shows nephritis and deafness; patients with these symptoms are sometimes suspected of having AS. Moreover, the pathological findings of Pierson syndrome frequently include lamellation of the GBM, as revealed by EM. Moreover, some cases of Pierson syndrome with missense variants in the *LAMB2* gene tend to show milder phenotypes involving later onset of ESRD [19]. These clinical features resemble those of AS. Cases with gene variants in *LMX1B* (nail–patella syndrome), *PAX2* (renal coloboma syndrome), or *MYH9* (MYH9 nephropathy) also show lamellation of the GBM, so they should be included in the gene list. Most mutations detected in AS are novel variants, because there are no mutational hotspots among the three genes. When we fail to detect pathogenic variants by means of targeted sequencing, we must proceed to the next step of detecting copy number variations (CNVs) and deep intronic pathogenic variants. Our gene screening strategy for AS is as follows: Step 1: targeted next-generation sequencing with a custom disease panel; when pathogenic variants are detected, they are confirmed by Sanger sequencing. When we fail to detect mutations by Step 1, we proceed to Step 2, which involves conducting pair analysis comparing NGS data from patients and normal controls, to screen for CNVs. When pair analysis shows the possibility of CNVs in any of the three genes, we proceed to detecting CNVs by multiplex ligation-dependent probe amplification (MLPA). When we fail to detect pathogenic variants with Steps 1 and 2, we proceed to Step 3: reverse transcription–polymerase chain reaction (RT-PCR) of mRNA and direct sequencing to detect aberrant splicing due to intronic or exonic variants. With these three steps, we can detect pathogenic variants in any one gene for more than 90% of cases clinically suspected of being AS [7, 12, 14, 20]. With Step 2, we recently detected CNVs in *COL4A5* in AS cases [21]. Pair analysis also revealed a case with clinically suspected AS because of ESRD and hearing loss, which had CNVs in the *EYA1* gene, and diagnosed this as BOR syndrome [21]. With Step 3, we have identified deep intronic variants that lead to cryptic exon insertion by creating novel splicing sites [12, 20]. We have also identified aberrant splicing caused by a so-called “silent variant,” an exonic single-nucleotide substitution that does not change the coding amino acid [22].

## Genotype–phenotype correlation

Three reports have been published analyzing the correlation between genotype and clinical picture based on large-scale data in male XLAS patients; we briefly summarize them here.

1. In 2000, Jais et al. analyzed 401 male XLAS patients from 195 families. The results showed that the median age at developing ESRD overall was 25 years; 90% of patients had developed ESRD by the age of 40. Genetically, the frequencies of progression to ESRD by the age of 30 were (1) 90% in cases with wide deletions, nonsense mutations, or frameshift mutations; (2) 70% in cases with splice site mutations; and (3) 50% in cases with missense mutations. This report showed for the first time that the kidney prognosis differed significantly depending on mutation type [8].
2. In 2002, Gross et al. conducted a meta-analysis of 267 men with XLAS, including cases that had been previously reported. They classified genetic mutations into the following three groups and evaluated their renal prognosis: (1) large rearrangements, premature stops, frameshift mutations, mutations of splice donor sites, or mutations in the NC domain (truncated protein group) were associated with the development of ESRD at the median age of 19.8 years; (2) missense mutations of glycine in exons 21–47, in-frame mutations, and mutations involving the splicing acceptor site (altered protein structure group) were associated with the development of ESRD at the median age of 25.7 years; and (3) missense mutations of glycine of exons 1–20 were associated with the development of ESRD at the median age of 30.1 years [23].
3. In 2010, Bekheirnia et al. analyzed 681 male patients with XLAS from 175 families. The results were as follows: The median age of developing ESRD was (1) 37 years with missense mutations, (2) 28 years with splice site mutations, (3) 25 years with truncating mutations, and (4) 22 years with both large- and small-deletion mutations. Furthermore, the position of mutations closer to the 5′ end tended to show earlier progression of ESRD; in the same analysis, limited to only missense mutations in glycine, there was no correlation between location and severity. In these respects, the results differed from those in the report by Gross et al. [24].

Regarding female XLAS cases, two previous reports showed no genotype–phenotype correlations [7, 9]. It has long been suspected that an uneven pattern of X-chromosome inactivation (i.e., skewed X chromosome inactivation) would influence the severity of XLAS in females [25]; however, no study has systematically proven the correlation between a skewed X pattern and clinical severity in female XLAS. In our experience, X-chromosome inactivation pattern analysis cannot predict kidney prognosis in female XLAS cases (manuscript in preparation).

For ARAS, two previous reports discussed the genotype–phenotype correlation in this condition.

1. In 2013, Storey et al. reported that patients with truncating mutations in at least one allele tended to show early onset of renal failure, compared with patients without truncating mutations [13].
2. In 2014, Oka et al. (our group) reported that no genotype–phenotype correlation was observed in ARAS cases, even when the patients were divided into two groups, similar to those of Storey et al. However, some cases that exhibited missense mutations in at least one allele showed milder phenotypes, with full expression of α5 on GBM [12].

In ADAS, no genotype–phenotype correlations have been observed thus far. Even within one family, clinical severity differed significantly and some individuals developed ESRD, whereas others possessing the same mutation showed no urinary abnormality [14].

### Mechanisms of onset of atypical mild phenotypes in male XLAS

It has been reported that some male XLAS cases show atypical mild phenotypes of late-onset ESRD. Based on previously reported results, it has been clarified that, for the following reasons, XLAS male patients may exhibit a mild clinical picture.

1. Cases with missense mutations

In all large-scale studies that analyzed genotype–phenotype correlations, it was reported that the age at which ESRD developed was significantly delayed in cases possessing missense mutations, as described above [8, 23, 24]. We have reported a male patient with very mild XLAS who had a missense mutation (p.Gly1000Val) in the *COL4A5* gene and only showed hematuria at the age of 38 years [26]. We have also encountered a 40-year-old male with the same mutation who only showed hematuria (unpublished data). Thus, some missense mutations can lead to extremely mild phenotypes.

2. Cases with in-frame deletion mutations

For mutations involving deletion of a multiple of three nucleotides, the fact that three bases code one amino acid means that this mutation does not significantly influence the subsequent amino acid sequence (i.e., no frameshift occurs). Such mutations are called in-frame deletion mutations. Gross et al. reported that, when an in-frame mutation occurs, the age at which ESRD develops is significantly later than in cases with frameshift mutations [23]. In addition, Jais et al. classified in-frame deletions with deleted nucleotides numbering from 3 to 18 bases into a missense mutation group and analyzed them, because they showed mild phenotypes [8]. We also have reported two families with mild disease phenotypes, the members of which progressed to end-stage renal failure in their 60 s and 40 s due to deletions of 9 and 36 bases, respectively [17].

3. Cases with splice site mutations result in in-frame deletion at the transcript level

Furthermore, we recently reported a male with a deletion of 105 bases at the mRNA level due to a splice site mutation, in whom ESRD had not developed at the age of 47 [20]. This case showed an in-frame mutation at the mRNA level, indicating that mutation at a splice site may be associated with a mild clinical picture. Recently, we published the results of a large data analysis comparing splice site mutations, with or without in-frame deletion, at the transcript levels; we revealed that renal prognosis differs significantly, and that ESRD developed 9 years later in the in-frame deletion group [27].

4. Somatic cell mosaicism in proband cases

**Fig. 4:**
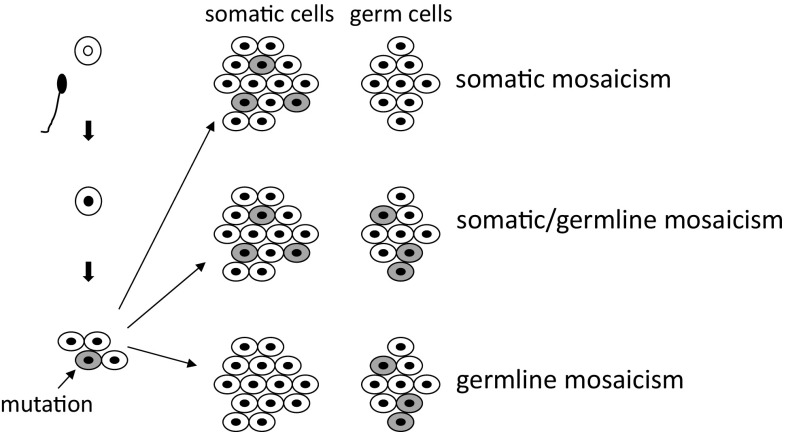
Schema of genetic mosaicism. When gene mutation occurs after repeated cycles of cell division in fertilized eggs, cells with a normal gene and cells with an abnormal gene coexist; this is known as mosaicism. When this status occurs in somatic cells, it is recognized as somatic mosaicism; in gonadal cells, it is known as germline mosaicism

Mosaic mutations involve the presence of two cell populations with different genotypes in one individual, which have developed from a single fertilized egg (Fig. 4). Probands in cases of hereditary diseases can sometimes develop to retain mutants at somatic cell division failure. When this gene mutation is inserted after repeated cell division in fertilized eggs, cells with a normal gene and cells with an abnormal gene coexist; this is known as mosaicism. When this status is observed among somatic cells, it is recognized as somatic mosaicism. We previously reported a male patient with mild XLAS who had a mutation with somatic mosaicism [28]. Recently, we reported a tendency for an association between variant allele frequency and severity of renal symptoms in four men with XLAS with somatic mosaic variants [29]. Variant allele frequency was analyzed by ultra-deep sequencing with NGS. One of the cases, whose two daughters exhibited typical findings of female XLAS, was completely asymptomatic.

5. A case with splice site mutation, in which both normal mRNA and aberrantly spliced mRNA are produced in renal tissue during transcription

With splice site mutations, exon skipping is usually observed. However, incomplete mutation of the splice site may produce both normal and abnormal kidney mRNA, leading to milder phenotypes. This mechanism has been reported in another inherited disease, galactosialidosis [30]. In this disease, the IVS8 + 9C > G variant in the CTSA gene produces abnormally spliced mRNA with a low level of normal mRNA, which leads to a milder phenotype in the patient. A similar mechanism was also observed in a male XLAS case with a mild phenotype. We recently reported a mild XLAS case that exhibited only hematuria and very mild proteinuria despite being 22 years; the patient possessed a deep intronic mutation that produced both normal and abnormal mRNA in the kidney [20].

6. Cases with α5 expression in GBM

In a typical XLAS male patient, because of abnormality of the *COL4A5* gene, expression of α5 is not recognized at all in GBM. However, the expression of α5 was confirmed in 29% of male XLAS patients; in those α5-positive cases, it was found that the age at which urinary protein was exhibited was significantly older, the amount of urine protein was significantly lower, the age of end-stage renal failure was also significantly older (37 years vs. 24 years), and the rate of hearing loss was also significantly lower. Thus, it was shown that α5-positive cases may exhibit a mild clinical picture [17]. Similar results were also recently published by a different group [31]. These cases with α5-positive expression in GBM possessed non-truncating (missense or in-frame deletion) mutations or somatic mosaic mutations in *COL4A5* [17].

## Treatment

There is currently no radical therapy for AS; treatment is only performed for the purpose of delaying progression to renal failure using nephroprotective drugs. A randomized controlled trial (RCT) is underway in Germany (EARLY PRO-TECT ALPORT study) for confirming nephroprotective effects. Two RCT trial results have been previously published examining reduction of urine protein levels by angiotensin-converting enzyme inhibitors (ACEIs) and angiotensin receptor blockers (ARBs) in AS [32, 33]. One compared losartan (ARB) and placebo, while another compared enalapril (ACEI) and losartan; both RCTs showed urine protein-reducing effects in both ACEI and ARB. A large retrospective study reported that ACEIs have the effect of delaying the progression to ESRD in AS [34]. Recently published expert guidelines recommend that male patients initiate treatment with ACEI from the time of diagnosis with AS [35]. Meanwhile, in female cases, it is recommended to start treatment at the time when urinary protein is detected, in addition to urine occult blood [35]. For ARAS, ACEI should be started at the time of diagnosis regardless of gender and, for ADAS, it should be initiated at the time when urinary protein is detected. No clinical trial has been performed comparing single (ACEI or ARB) and double block (ACEI and ARB); theoretically, double block can show a stronger renoprotective effect, and is already applied in clinical settings. It was previously reported that cyclosporine treatment for AS significantly reduced urine protein levels and showed renoprotective effects in long-term follow-up [36, 37]. However, three recent reports showed that, although cyclosporine had strong effects for reducing urine protein, it accelerated interstitial fibrosis and had no renoprotective effects [38–40].

Regarding kidney transplantation, good results have been obtained compared with those in other kidney diseases; however, in 2–5% of cases, anti-GBM antibody is produced, leading to rapid graft loss [41]. There is no curative therapy for hearing impairment; only symptomatic treatment is available, such as the use of a hearing aid.

Some new drugs have entered clinical trials, such as bardoxolone methyl (Phase II/III) and RG-012 (effect on microRNA-21 interference, Phase II). Other therapies that have thus far been reported to show effects by in vivo and in vitro trials are as follows: (1) treatment with stem cells [42] and (2) treatment to control intracellular signal transduction (USAG-1) [43]. Further expected treatments are as follows: (1) nonsense read-through therapy [44] and (2) exon skipping therapy with antisense oligonucleotides or chemical compounds [45, 46]. Notably, our group is working to develop exon skipping therapy using antisense oligonucleotide (ASO). ASO will bind to the exonic splicing enhancer region and disturb the targeted exon at the point of splicing; this will lead to exon skipping. As described above, when the skipped exon nucleotide number is a multiple of 3, it change the severe phenotype with nonsense mutation to a milder phenotype with an in-frame deletion. We have already obtained good results in vitro and are proceeding to an in vivo trial with an animal model.

## The border between TBM and AS

An issue of particular interest among experts in the field of AS is the differential diagnosis between AS and TBM. AS has generally been defined as progressive nephritis, sensorineural hearing loss, and specific eye abnormalities; TBM is generally defined as hematuria with/without mild proteinuria, with diffuse thinning of GBM that does not result in renal failure. There remains no clear delineation between the two diseases and leading clinical nephrologists are quite confused. Most heterozygous carriers of variants in *COL4A3* or *COL4A4* or some females with heterozygous mutations in *COL4A5* show only hematuria without proteinuria; those cases are not diagnosed with AS, according to its current definition. It is well-known that most cases with benign familial hematuria possess heterozygous mutations in either *COL4A3* or *COL4A4*, and most will not develop ESRD [47]. It is also well-known that, in cases with only hematuria without proteinuria, ESRD will never develop [9]. In EM findings, these cases typically show only TBM without lamellation of the lamina densa [7, 14]. In contrast, cases with ADAS begin to show proteinuria at the median age of 17 years; for females with XLAS, the equivalent age is 7 years. These findings indicate that there is no clear distinction between these two diseases, which presents difficulties for physicians when attempting to make a differential diagnosis. We have experienced some cases exhibiting only hematuria at a young age, which were diagnosed with TBM; these patients then missed annual check-ups and developed ESRD at approximately 60 years of age (personal experience). ACEI can delay the age of developing ESRD, even in male cases of XLAS with the most severe phenotype. This indicates that ACEI can remarkably delay the age of ESRD development for cases with much milder phenotypes of ADAS. As experts in this field, we are responsible for ensuring that, in AS cases, the opportunity to begin treatment by ACEI is not missed. Diagnosis of TBM tends to underestimate the risk of progressive kidney disease. Therefore, some experts have recently asserted that patients with hematuria, TBM, and heterozygous mutations in *COL4A3* or *COL4A4* should be classified as cases of autosomal AS. In addition, all females with XLAS should also be classified as cases of XLAS. These changes of definition of AS may eliminate TBM nephropathy as a diagnostic entity [48].

## Conclusion

Recent advances in genetic analysis have provided us with a deeper understanding of the pathophysiological mechanisms of inherited kidney diseases. Based on precise genetic information, we came to understand the mechanisms of onset of milder phenotypes in AS. As experts in this field, we must devote our energies to creating a system that allows patients with AS to be diagnosed at an early stage and to start appropriate treatment. For this reason, we need to change the definition of AS to include all cases with nephropathy caused by *COL4A3, COL4A4*, or *COL4A5* gene variants and eliminate the disease entity of TBM; this will allow patients to avoid missing the opportunity to start appropriate treatment as early as possible.
